# Unveiling the Flowers: The Views of Female Victims of Rape on the Care Offered in a Teaching Hospital

**DOI:** 10.3390/ijerph22081264

**Published:** 2025-08-13

**Authors:** Melissa de Oliveira Araújo, Karla Patrícia Cardoso Amorim

**Affiliations:** 1 Postgraduate Program of Public Health, Federal University of Rio Grande do Norte, Natal 59078-900, Brazil; 2 Department of Medicine, Federal University of Rio Grande do Norte, Natal 59078-900, Brazil; amorimkarla@yahoo.com.br

**Keywords:** rape, sex offenses, domestic violence, women’s health services, sexual trauma, legal abortion

## Abstract

This study aimed to analyze the perception of women who were victims of rape about the care provided at a teaching hospital located in the northeast of Brazil. A qualitative study was conducted with seven women. The interviews took place in a trustworthy and confidential environment in the presence of a psychologist. The data were analyzed through content analysis, evidencing the women’s perceptions. The sample was achieved by convenience sampling and was homogeneous, with a profile of women who were young, white/black, and single, with low education and a history of sexual violence. Positive views about the care provided at the hospital included its status as a reference center, motivating women to seek health services. The legitimization of women’s rights, effective care, and the possibility of having a legal abortion were also mentioned. The challenges reported included the need for a prepared health team and more humanized and multidisciplinary care. Women who had suffered rape sought medical assistance for support, guidance, and a humane approach to understanding their rights, often finding a compassionate response from the teaching hospital staff. Despite advances in care, challenges remain, such as perceived judgment regarding legal abortion, highlighting the need for policies that better meet the needs of victims.

## 1. Introduction

Violence against women is described as “any act of gender-based violence that results in, or is likely to result in, physical, sexual or mental harm or suffering to women, including threats of such acts, coercion or arbitrary deprivation of liberty, whether occurring in public or private life” [1,2]. Sexual violence (SV) comprises any sexual act involving violence, coercion, and unwanted sexual comments or assaults, regardless of the relationship with the victim [3].

The United Nations (UN) warns that throughout their life, one in three women—around 736 million people—are subjected to sexual violence by their partner or non-partner [4].

Notes from the United Nations Children’s Fund (UNICEF) reveal that Sub-Saharan Africa has the highest number of victims, with 79 million girls and women affected (22%), followed by 75 million in East and Southeast Asia (8%), 73 million in Central and South Asia (9%), 68 million in Europe and North America (14%), 45 million in Latin America and the Caribbean (18%), 29 million in North Africa and West Asia (15%), and 6 million in Oceania (34%) [5].

A total of 66,020 rapes were reported in Brazil in 2021, representing an increase of 4.2% compared with the previous year. About 75.5% of the victims were vulnerable people not able to consent, 61.3% were under 13 years old, and 79.6% knew the rapist. The state of Rio Grande do Norte (RN) reported 494 cases of rape in 2021 in which the victim was a woman, evidencing the need to evaluate public policies in this field [6].

Regarding the vulnerability that is often inherent in victims of sexual assault, vulnerability is understood as a condition of being exposed to risks, harm, or adversity, whether in physical, emotional, social, or economic aspects. For women who have suffered sexual assault, one of the most common vulnerabilities was economic [3].

The occurrence of rape is a public health issue due to the risk of leading to unwanted pregnancy, unsafe abortion, sexually transmitted infections, sexual dysfunctions, infertility, pelvic pain, post-traumatic stress disorder, depression, panic disorder, and suicide, among other physical and psychological distresses [2,6]. Unwanted pregnancy resulting from rape has psychological, social, and biological repercussions. Furthermore, most women do not have access to health care services to obtain an abortion, even though public health care is free in Brazil, and there is a lack of trained professionals and adequate infrastructure. Additionally, information about its legality and rights is often withheld when women seek care [7,8].

The search for help after an experience of sexual violence is often crossed by barriers that reflect the depth of the trauma experienced. Among the most cited by victims are the fear of blame and moral judgment, fueled by a culture that still holds those who suffer violence responsible [7].

Shame also emerges as a central obstacle that is reinforced by the intimate exposure required to report the incident. Many victims fear not being believed, especially when the aggressor is in a position of power or emotional closeness. Institutional revictimization, through insensitive procedures, repetitive reports, and prolonged investigations, increases suffering and discourages reporting [9].

These fears are intensified by the lack of safe support networks and the absence of effective public policies. To break this cycle, it is necessary to build welcoming environments, based on empathetic listening and the guarantee of rights, where the victim is seen as the subject of her own narrative and reconstruction [10].

In Brazil, balancing the health needs of female victims of rape and existing public policies is difficult. Moreover, health services for this population tend to be inefficient and fail to ensure broad access, humanized assessment, and comprehensive care [9,10]. Policies must be created to qualify the care of victims of rape; therefore, their perspective on the care offered in health services needs to be evaluated.

This study was designed based on a direct approach to victims of sexual assault who suffered rape, aiming to build a more humanized perception of each life story marked by pain and suffering. Questions guiding our investigation encompassed the following: How is the care for female victims of SV operationalized? How does the care offered to these women impact their lives? What are their perceptions of the care offered? Thus, this study aimed to analyze the perception of female victims of SV on the care offered in a teaching hospital.

## 2. Literature Review

Violence against women is the result of a historical construction that reinforces stereotypes of female submission, exposing them to various forms of aggression [11]. This construction, deeply rooted in patriarchal structures, is sustained by discourses and practices that normalize gender inequality [12]. Despite the efforts of the UN since the 1950s to combat this violence, discussions often face cultural and social resistance, highlighting how systems of oppression renew and adapt to societal changes, keeping women in vulnerable positions [13].

In Brazil, public policies and feminist movements have driven significant progress, such as the establishment of the National Council for Women’s Rights, Specialized Police Stations for Women’s Assistance, and the Maria da Penha Law, which introduced pioneering mechanisms to prevent and combat domestic violence [14,15,16]. These milestones reflect not only the fight for equality but also the recognition of violence as a structural issue requiring coordination among various social and institutional spheres [15].

In the field of public health, progress has been made by addressing gender-based violence as a health issue, with the creation of the Comprehensive Women’s Health Care Program in 1983 and the National Policy for Comprehensive Women’s Health Care in 2004 [16]. Conferences like the 1986 event consolidated the Unified Health System (SUS), emphasizing women’s health as a fundamental right. Moreover, the 1994 Belém do Pará Convention underscored violence against women as a human rights violation, emphasizing the need to overcome discriminatory practices rooted in patriarchal structures [17].

Since the 1980s, debates on violence against women have adopted the category of gender rather than focusing exclusively on women. This shift enabled a broader analysis of the social constructions of femininity and masculinity, highlighting how these categories intersect with race, class, and other dimensions. Among the leading theories on the topic, Marilena Chauí discusses the ideology of male domination and female complicity rooted in a lack of autonomy; Heleieth Saffioti connects patriarchy with capitalism and racism, exposing unequal power relations; and Maria Filomena Gregori advocates replacing the term “victims” with “women in situations of violence”, emphasizing the relational dynamics underlying these experiences [18,19].

Although the concept of patriarchy is often criticized for its historical rigidity, the use of the gender category allows for a more dynamic approach, encompassing race and class issues. Even so, male domination continues to restrict women’s freedom and justify various forms of violence, as evidenced by high femicide rates and the persistent impunity of aggressors. This reality reveals how patriarchy manifests in both public and private spheres, perpetuating female subordination [20].

Sexual violence, recognized as a severe public health issue, affects women of all ages and social classes. Studies indicate that approximately 35% of women worldwide experience physical and/or sexual violence in their lifetime, often perpetrated by intimate partners. This type of violence, which violates sexual and reproductive rights, frequently prevents victims from seeking medical assistance due to fear, shame, or coercion, further deepening gender inequalities [21].

In Brazil, sexual violence reflects structural inequalities of class, race, and gender, exacerbated by the feminization of poverty, which exposes women to greater vulnerability. Pregnant women often face complications resulting from violence, revealing how control over their bodies is exercised in both interpersonal and institutional contexts. Additionally, shortcomings in healthcare services, such as the lack of adequate protocols and institutional negligence, hinder victim care, perpetuating cycles of violence and exclusion [18].

Although underreporting remains a challenge, legal and regulatory advances have improved victim care in Brazil. The first guideline, issued by the Ministry of Health in 1998 and revised later in the years 2004, 2012, and 2015, formalized measures for comprehensive and multidisciplinary care, including the guarantee of legal abortion without the requirement of a police report. However, laws mandating the notification of cases to police authorities have sparked criticism, as they may increase victims’ vulnerability, particularly in contexts where institutional trust is limited [11,13,19].

Comprehensive care for women requires intersectoral coordination, which is essential to prevent revictimization and ensure quality support. The National Policy for Comprehensive Women’s Health Care addresses sexual violence with an emphasis on humanization, quality of care, and user empowerment. Nevertheless, challenges such as the lack of sector integration and an excessive focus on pathological aspects hinder progress in addressing sexual violence within SUS [22].

Sexual violence not only compromises women’s physical and emotional health but also affects their education and quality of life. Thus, there is an urgent need to implement more effective public policies that are sensitive to the social and structural complexities perpetuating violence against women. This transformation demands a collective commitment to dismantling the cultural and institutional foundations that sustain gender inequality, ensuring women’s right to dignity and autonomy over their bodies and lives.

## 3. Materials and Methods

We conducted a qualitative, exploratory, and analytical study to understand the perception of female victims of SV on the care offered in a teaching hospital. This social research within the health area proposes a dialectical perspective to understand the complexity of relationship systems, considering the exterior (apparent explanations about a phenomenon) and interior (explanations that emerge from the in-depth investigation of reality) of the constitutive bases of the phenomena [23]. Qualitative research uses words to demonstrate the everyday speech found in social relations and interviews to identify the representations of groups determined by their historical–structural conditions [23,24].

The hospital where the data were collected is a public teaching hospital in the state of Rio Grande do Norte (Brazil). This institution is a reference in the care of women who are victims of sexual violence. The hospital began offering specialized care for this population in 2001 [25] in the Obstetric and Gynecological Emergency Service. The service operates 24 h a day, with a multidisciplinary and interdisciplinary reference team, is responsible for the organization, assistance, and technical guidance of other professionals, and includes a doctor, nurse, social worker, and psychologist.

The hospital adopts the guidelines and directives of the Ministry of Health to support legal abortion. In the first trimester of pregnancy, the recommended methods for pregnancy termination include intrauterine aspiration (manual or electric), pharmacological abortion, and dilation and curettage. The choice of method is determined by the availability of structure at the hospital, the patient’s preference, and the analysis of the risks and benefits of each procedure. From the second trimester of pregnancy, medical abortion is the only authorized method. After the expulsion of the fetus, this procedure can be combined, when necessary, with curettage or uterine aspiration. The termination of pregnancy through microsurgery or microcesarean should only be performed in exceptional cases [26].

Study participants included female victims of SV aged ≥18 years who received care at the hospital in 2021. The sample was conveniently selected via personal invitation letters from the principal investigator when the women were admitted to the hospital emergency room for health care due to SV. There was no phone contact or subsequent invitations in order to avoid revictimization, pain, and suffering. Women with mental disorders were excluded as this could add distress to their psychological conditions. We adopted the data saturation criterion to determine the sample size, and participants were given flower names to ensure anonymity. Data were collected between 13 May and 30 September 2021, using semi-structured interviews conducted in a private room at the hospital by the main researcher who is a social worker at the hospital, with the assistance of a hospital psychologist to provide emotional support to the participants.

The researcher was in the field for four days a week, with a daily workload of twelve hours. All patients who attended the hospital during this period and met the eligibility criteria participated in the research until we reached the theoretical saturation of the data.

Interviews lasted about 40 min and followed a script (Appendix A) with open-response items in order to not limit freedom of speech and spontaneity [27]. The researchers did not have any previous interactions or relationships with the participants. Questions encompassed sociodemographic information and evaluation of the care offered in the hospital. A voice recorder was used with consent to facilitate data collection; therefore, it was not necessary to repeat any interview.

The data were analyzed in four stages using Laurence Bardin’s Thematic Content Analysis [28]. First, the interviews were transcribed in full, generating a textual corpus. In the second stage, a pre-analysis of the corpus was conducted through skimming, document selection, reformulation of objectives, and formulation of indicators.

Laurence Bardin’s Thematic Content Analysis is a method of interpreting qualitative data to identify relevant themes in texts. It occurs in three main stages: pre-analysis, exploration of the material, and processing of the results. In pre-analysis, the corpus is selected, hypotheses and objectives are formulated, and indicators are defined. In exploration, the text is fragmented into units of analysis and organized into thematic categories. Finally, the results are described, interpreted, and related to the theoretical framework, allowing for an in-depth understanding of the content analyzed [28]

In the third stage, individual analysis of interviews identified terms and recurring themes considering the theoretical framework. The focus encompassed the subjectivity of participants and care received post-violence, aiming to codify registration and context units to formulate broader categories.

In the last stage, data were further analyzed through inferences and critical, reflexive, and interpretative analyses. Two tables were created to relate the information from interviews and the variables of the analytical model using thematic categories, units of meaning, and synthesis of speeches.

This study was approved by the research ethics committee, protocol number 4,698,570. All participants were informed before the interview about the purpose of the research, data collection methods, the position of the interviewer, and the possibility of declining participation in the study at any time. Written consent to participate was obtained from all participants.

## 4. Results

Throughout the research period, the hospital treated thirty-one female victims of rape. Eleven were adolescents, one was a child, and one had a mental disorder. These participants were excluded from the study. Seven women participated in the study. Given the homogeneity of the sample in terms of education level and occupation, we reached theoretical saturation, and it was not deemed necessary to expand the recruitment. Five were admitted for legal abortion and two were admitted for prophylaxis of sexually transmitted infections and emergency contraception administration. The participants were between 22 and 58 years old, of whom three were white and four identified themselves as black. Three of them went to college, but only one completed their studies. The main professional occupation was nursing technician (n = 4). Six participants had formal professional occupations with a monthly income of BRL 400.00 (approximately USD 80 in 2021) to BRL 2000.00 (approximately USD 327.80 in 2021). Their characteristics are described in Table 1.

Regarding the five pregnant women who sought care at the hospital, three were nursing technicians and opted for a legal abortion. Two of these women were in the early stages of pregnancy (6 and 8 weeks) and did not seek other healthcare services before seeking care at the hospital. The third woman, at 11 weeks of gestation, was referred to the hospital by a private medical office.

The results of the interviews were organized into three thematic categories, namely, (I) flowers wounded by violence—the search for care; (II) cultivating care—flowers wounded in the health service; and (III) unveiling the flowers—contributions to improving care.

### 4.1. Flowers Injured by Violence—The Search for Care

This category addresses the experience of female victims of violence seeking health care, highlighting the emotional, social, and institutional implications faced during this process. Many of these women, even in situations of extreme pain and vulnerability, demonstrated the strength to seek medical support, driven by the desire to overcome the trauma and take care of themselves. However, they faced significant challenges, such as delays in accessing effective care due to a lack of clear information, institutional barriers, and the absence of prophylaxis or emergency contraception methods.

“*I just wanted to put an end to it*” (Rose).

“*I knew I needed to go to a hospital*” (Hydrangea).

“*I think the help, the support to solve this issue, I didn’t know where to look anymore. I didn’t want to do it alone because I didn’t want to put my life at risk*” (Tulip).

“*It’s really bad to have a child from a person you don’t even know who is, right? Because when it’s from a husband or a boyfriend you know what to do, and how it was, but having a child of a person you can’t even imagine…*” (Sunflower).

Pregnancy resulting from violence was particularly difficult for some women, generating a strong desire to terminate the pregnancy. This emotional conflict was exacerbated by the difficulty of creating an emotional bond with a child conceived in such traumatic circumstances, especially when the father was unknown. In addition, the lack of knowledge about available services and legal rights made it difficult to make informed decisions, increasing the vulnerability and suffering of these women. The need for psychological support, protection, and clear information emerged as one of the main aspects reported, showing that the lack of adequate guidance can prolong despair and increase the negative consequences of the violence suffered.

“*I wanted to terminate the pregnancy*” (Orchid).

“*I even thought about killing myself, so desperate I was; not knowing the time was passing, it was passing, because if I knew that I had this type of care in the hospital, I would have sought care it much earlier*” (Sunflower).

“*It was to protect myself on what might happened to me with that act and to have orientation of what I could do, what my rights were, in that state of vulnerability*” (Amaryllis).

Another significant aspect was the impact of shame and stigma, which led many women to isolate themselves and avoid seeking help, especially regarding the idea of terminating their pregnancy. This feeling was compounded by the fear of suffering institutional violence, such as judgment or criticism from health professionals. Despite this, positive experiences of acceptance, without judgment, were described as essential to mitigate these fears and provide emotional relief.

*“I was desperate, I didn’t want to have [a baby] and my family even thought of me having and giving away, but I didn’t want to give away either, because I already have my four children, so I didn’t want to have one to give away, because of this”* (Sunflower).

*“No, just from my story of what I went through, it’s normal to be ashamed…”* (Hydrangea).

*“How was I going to get to the hospital asking to have an abortion? That’s what I thought”* (Sunflower).

*“I just felt like this, because I have been crying, embarrassed. Although they said in the clinic that I would be welcomed here, but all the time I came with anguish, apprehension, because my fear was to get here and people condemn me, criticize me, but no, this did not happen here”* (Azalea).

The results highlight the need to improve the communication, support, and accessibility of health services for female victims of violence. Institutions that offer a safe, empathetic environment with clear information about rights and procedures are essential to reduce the impact of trauma and promote appropriate care. The creation of public policies that ensure psychological support, access to legal abortion, and sensitive protocols to assist these women is essential, as the lack of guidance and support prolongs suffering and increases the risk of serious psychological and physical consequences.

### 4.2. Cultivating Care—The Injured Flowers in the Health Service

This category addressed, in a compelling manner, the experiences of female victims of violence who sought care in health services, focusing on aspects of reception, support offered, and challenges faced during the process. The central theme is the relationship of women with the health system as they try to deal with the trauma of the violence suffered and make decisions about the necessary care, including legal abortion.

Many women reported feelings of safety and acceptance when receiving care, highlighting the importance of an empathetic approach on the part of professionals. This acceptance provided relief in moments of great vulnerability, especially when faced with the fear of being judged or criminalized.

“*I felt good, because I thought that if I came and asked something like this I would be arrested*” (Sunflower).

“*I felt safe*” (Sunflower).

Some women praised the speed and efficiency of the service, describing the support they received as essential in dealing with the trauma of violence. These positive responses helped to create a space of trust and alleviate concerns about possible complications, such as the transmission of sexually transmitted diseases.

“*Wonderful, everyone assisted me very well, they were very supportive, and I have no complaints about anything, just thank for the solidarity of each one of you*” (Hydrangea).

“*[…] we are in a state of vulnerability, of not knowing what to do. We end up receiving any help that comes, this happens a lot. So, as I did not have much knowledge and I was trusting that here could really help me, specially to prevent a disease, because trauma is something that we can work on, but if I had any disease, an AIDS or something like that, I would have had to deal with it for the rest of my life*” (Amaryllis).

This category also highlights the need to increase access to information about women’s rights in situations of violence. Some women felt that the health system should provide clearer and more accessible guidance from the outset, avoiding the feeling that rights are “discovered” only through the advice of others.

“*[…] I knew this [the service] because someone knew about it and informed me. But as a woman, I have no right to know? Couldn’t this [information] already be passed on to us in another way? The access to this information*” (Amaryllis).

For some women, the care involved having a legal abortion, a delicate moment that required detailed explanations and emotional support. The clarity of the guidelines and the empathy of the professionals helped to alleviate fear and despair, especially in cases of sexual violence that led to pregnancy.

“*They explained to me how the procedure was going to happen. I was afraid because I had never been through this […] for me it’s kind of solving my problem because I wasn’t sleeping. I was desperate with the situation [rape]. I was in, I didn’t know what to do*” (Sunflower).

“*They received my demand and explained step by step of the procedure, even said that it would take a while […], but then I understood the procedure [abortion] because I had to go to the commission*” (Azalea).

Despite the positive experiences, some women reported moments of insecurity and embarrassment, which were usually associated with the attitudes of professionals who could have been more careful when addressing sensitive issues. These situations reinforce the need for ongoing training for multidisciplinary teams in order to ensure that all care is guided by respect and empathy.

“*Everyone assisted me very well. I was very nervous, crying. They asked me to calm down that everything was going to be okay and everything I said here was going to be confidential, no one was going to criticize me, because I came and asked, ‘Is anyone going to criticize me because I’m doing this?’, ‘Not at all. You’re a woman and you know what you want, you have the right to do it.’ I said ‘okay’—that’s when I felt more relieved. But I was desperate, I came all the way here crying, thinking about how they would look at me when I got here, but so far everything is fine*” (Azalea).

“*I felt a little embarrassed now, in this last visit with the physician when she was questioning on dates, period […] I was frightened by the possibility of not deferring [abortion]*” (Tulip).

*[…] I felt like I had done something wrong, very wrong, at some point, and I know I did not, with absolute certainty*” (Sunflower).

Seeking healthcare services is a crucial step for women who have suffered violence, as it can determine not only the resolution of immediate medical issues but also the relief of emotional trauma and the restoration of a sense of control over their lives. The experiences reported by the women highlight the importance of humane and efficient care, with clear guidance on rights and available procedures. However, situations of insecurity or embarrassment reveal the need for more robust guidelines for multidisciplinary teams, ensuring that care is universal and free from judgment. In addition, legitimizing women’s rights and expanding access to information about available services are essential to strengthen trust in the healthcare system and promote patient well-being.

### 4.3. Unveiling the Flowers—Contributions to Improving Care

This category discusses contributions to improve care for female victims of sexual violence, highlighting gaps, good practices, and suggestions for improving health services. It addresses the experience of patients and the factors that directly impact the reception and quality of care provided.

There is criticism of the lack of dissemination and communication about the services available to women in situations of violence. This limits access and the possibility of seeking help in an informed manner. Women emphasized that access to information should be guaranteed and promoted broadly. In addition, it was noted in the speeches that there is an emphasis on the support role of the health service, recognizing that bodily autonomy belongs to the woman, and not to the professionals.

“*I thought it lacked communication to society [about the service]. I knew this because someone else knew it and informed me, but as a woman I have no right to know? Could this not already be passed on to us in another way? The access to this information*” (Amaryllis).

“*When a woman decides to terminate a pregnancy in cases of rape, in cases of abuse, she has already decided that, you know, […] she needs support, you know*” (Sunflower).

“*Because the body does not belong to the physician, the body belongs to the victim and she needs to decide what she wants to do*” (Tulip).

Reports show that immediate care is essential, but a lack of awareness about available services can delay seeking help. This delay intensifies emotional distress and reduces the chances of preventing serious illness or consequences.

“*[…] I did not know what to do, I even thought about killing myself, so desperate I was; not knowing the time was passing, it was passing, because if I knew that I had this type of care in the hospital, I would have sought it much earlier*” (Sunflower).

The speeches highlighted the need to rethink the system since it needs to be interconnected, multidisciplinary, and interdisciplinary. It needs to be capable of offering continuous support, from emergency care to medium- and long-term monitoring, ensuring safety and comprehensive support for women.

The women emphasize the importance of continuous monitoring, including psychological support and gynecological care, as a way of dealing with trauma and regaining confidence in themselves and the health system.

The presence of professionals from different areas, such as social workers and psychologists, was highlighted as essential for humanized and comprehensive care, especially in situations of high vulnerability.

“*[…] we are in a state of vulnerability, of not knowing what to do. We end up receiving any help that comes, this happens a lot. So, as I did not have much knowledge and I was trusting that here could really help me, especially to prevent a disease […]*” (Amarilis).

“*If I could have outpatient support, for other women as well, because that’s very important. Many women go through it [rape] and just stay silent, they don’t come out. So always having a place giving support, welcoming in any way, because here I am feeling welcomed*” (Hydrangea).

“*I came to talk to the social worker, and it was very welcoming and humanized*” (Tulip).

The need for ongoing training of health professionals was highlighted, ensuring that they are prepared to deal with cases of sexual violence in an ethical, sensitive, and informed manner. This preparation is crucial to ensuring that women’s rights are respected and promoted.

“*I say that it [service] met my expectations […] I arrived here, and, at all times, we were very well assisted, welcomed. They explained to me everything that could have happened to me, step by step, that I was going to have assistance, also the medical care to ask for exams, having medications ready for that moment. So, I don’t have much to complain about what I experienced here. Since I have a closer contact, I think that in every moment I was well assisted and I really like this safety mainly because I went through a moment that I was unprotected from everything with that person (abuser), and here it isn’t like that, it changed the feeling I was having. I’m feeling peaceful*” (Amaryllis).

In summary, this category suggests that improvements in hospital services depend on strategies that promote the communication and dissemination of women’s rights, the development of integrated care networks, and the guarantee of humanized, agile, and continuous care. The formation of multidisciplinary teams and the ongoing education of professionals are essential to ensure adequate care and respect for women’s autonomy and reduce the negative impacts of sexual violence. In short, strengthening public policies and care protocols is essential to transform women’s experience in the health system and ensure effective support in times of extreme vulnerability.

The positive perceptions and challenges regarding the care provided in the hospital were extracted from the first two thematic categories, and contributions to improving care were extracted from the third thematic category (Table 2).

## 5. Discussion

This study analyzed the perceptions of female victims of sexual assault who were raped about the care provided at a teaching hospital. Although our findings showed that they sought care at a health service, female victims of rape often reported receiving poor information and difficulty accessing specialized services.

The participants’ statements demonstrated the distress they experienced when seeking health services. After finding them and feeling welcomed at the hospital, they received effective care. However, the women still reported being judged morally by health professionals and expressed the need for better psychosocial support.

Regarding the profile of the women treated, they had homogeneity in skin color, with most of them being single and low-income, with a minimum age of 18 and a maximum of 58 years. A similar profile has been described in other national studies, evidencing the influence of social conditions on cases of sexual assault [29,30]. Thus, considering the social vulnerability of this group, understanding how these women evaluate the quality of care offered by health services is important to provide equitable care [31].

Other relevant information retrieved from the interviews was that most participants had completed secondary and/or higher education and worked in the health sector, indicating that they knew where to seek care and were aware of the harmful consequences of SV. Professional occupation and education may explain why most women came to the hospital through referrals from other public services, evidencing an understanding of how they could find support within the health system [32].

Although some of these women knew how to navigate the health system to seek care and assistance, this is not the reality for many women in Brazil. In 2023, in the state of Rio Grande do Norte, 31.1% of the population aged 25 and over did not complete primary school, and 10% of the population of the same age had no formal education at all [33]. Illiteracy at age 15 and over affects 3.2% of white women and 6.7% of black women in Brazil and is progressively higher with age and lower income [34]. Access to timely care for this population, which is already more vulnerable to sexual assault and rape, becomes even more important [29,30,32].

Despite the suffering and the difficulty in finding effective and specialized care that provided prophylaxis and emergency contraception, women demonstrated interest and a continued need to seek health services. Seeking health services in different facilities is frequent among female victims of SV [19]. The exclusion of SV care as an institutional function demonstrates its invisibility within health services. Victims are then forced to look in unusual places until they receive dignified care. The lack of access to immediate care after rape may culminate in unwanted pregnancy or sexually transmitted infections and further emerges as a health demand [20,32].

Assistance to female victims of rape requires the attention of health professionals because victims commonly carry a distinct feeling from other forms of violence. As cited by the participants, feeling ashamed may accompany the care process, especially when it culminates in abortion [35,36]. Thus, the health service must be welcoming, secure, and protective and respect women’s rights and autonomy [31,32]. Our findings identified that participants felt welcomed, understood, and had their rights respected and legitimized by most professionals at the teaching hospital.

Nevertheless, some participants reported the need for more clarification about their rights. Women might have positively evaluated the hospital simply because they could receive assistance and not because they acknowledged their rights as being respected. This hypothesis is reinforced by the feeling of gratitude expressed by the victims for receiving care in a vulnerable time, a response pattern previously reported in the literature [35,36,37,38].

Women have the right to an abortion in cases of unwanted pregnancy resulting from rape [39]. Among all the participants, five women decided to take part in the procedure, and most were unaware that the hospital offered this service. The speeches evidenced a break in the expectations regarding legal abortion. When granting an abortion, the hospital opens an investigation to collect accurate information about the rape. This data collection is a crucial step for offering care for these women [39,40,41,42]. The desire for a quick procedure was confronted with institutional bureaucracies, which seemed too slow for those suffering [40,43,44].

Also, the women reported the feeling of distrust by the health professional during data collection. Attitudes of distrust during an initial assessment at a health service increase the sense of fear, criticism, judgment, and the fear of litigation due to their perception of performing an illegal procedure [30] and may culminate in institutional violence [31,45].

Regarding abortion, it is evident that women seem to distinguish between the care they receive from doctors, social workers, and psychologists, for example, showing that creating connections with victims of rape can be more challenging for some professional categories [29]. Even health professionals with established knowledge about the right to perform an abortion may adopt an investigative approach [7].

Therefore, standardized protocols to offer care for female victims of rape are required. Protocols must respect premises established by health policies and guarantee the right to dignified high-quality treatment, especially in vulnerable times [46]. This study demonstrated that health professionals need to be better trained to provide comprehensive care and mediate access to constitutional rights, care actions, referrals, and notifications in suspected or confirmed cases of violence, aiming to contribute to policies to prevent SV. Health teams should be qualified to welcome individuals and provide care aimed at physical, psychological, and social recovery without any discrimination that may lead to the interruption of care [47,48].

Although the hospital where data were collected has an outpatient clinic for the gynecological follow-up of children and adolescent victims of SV, the service is not available for adult women. The absence of follow-up for this population hinders the understanding of SV consequences in the medium term and the effectiveness of prophylaxis [49].

Also, we observed that social service and psychology professionals were unavailable in the 24 h care service, failing to enable multi-professional care for victims of rape. Therefore, depending on what time the victims seek care, the assistance is restricted to clinical assessment, which is not aligned with comprehensive care [50]. Unfortunately, the need for more specialized health professionals in this context is frequent [35,36]. Participants repeatedly reported the need for these professionals, highlighting the importance of receiving clarification about their rights and exposing a challenge to women’s health system networks [36]. It was evident that the low institutional adherence of qualified human and material resources in the hospital has the potential to fragment care and hinder access to health, weakening what is proposed by the Brazilian health care network [39].

A lack of information about the care offered by the hospital also demonstrates the fragility of the healthcare network [32,37,43]. The misinformation may lead to irreversible consequences for the health and well-being of women and may occur due to the difficulty women and professionals have in dealing with the theme of SV [47,50]. Misinformation is also accompanied by a lack of a sense of belonging, which demonstrates a disarticulation between public policies and the targeted population.

The healthcare network allows women to have different entryways, which must be organized to welcome and refer accordingly to the demands of each situation [38,45]. Health services must be integrated and should count on specialized professionals to optimize performance. The absence of protocols and specific flows of care corroborates with the non-institutionalization of the service, contributing to poor accountability of health professionals in specific cases and fragmenting care [49].

The study on care for female victims of sexual violence reveals several limitations that impact the quality of care provided. First, the homogeneity of the sample, composed of women with similar profiles in terms of skin color, marital status, and socioeconomic conditions, limits the generalization of the results to more diverse populations. In addition, the fact that the study was conducted in a single health institution may not reflect the reality of other units, which compromises the comprehensiveness of the conclusions. Another critical point is the lack of long-term follow-up of the women served, which makes it difficult to analyze the consequences of sexual violence and the effectiveness of the interventions carried out. The lack of specialized professionals, such as social workers and psychologists, in 24 h care also compromises the provision of comprehensive and multidisciplinary care.

Institutional bureaucracy, especially in relation to legal abortion, was identified as an additional source of suffering for victims who face slow processes in times of vulnerability. Misinformation, both on the part of women and health professionals, about the services available contributes to gaps in care. The fragmentation of the healthcare network, resulting from the lack of standardized protocols and specific care flows, is also a significant limitation, as it makes it difficult to hold professionals accountable and ensure continuity of care. In addition, some participants reported feeling morally judged or distrusted by healthcare professionals, which negatively impacted their experience of care.

In light of these limitations, several directions for future studies emerge. It is essential to investigate more diverse populations, considering race, social class, and cultural context, to better understand the different experiences of female victims of sexual violence. Comparative studies across different healthcare institutions can help identify good practices and areas that need improvement. A longitudinal follow-up will allow us to assess the physical, psychological, and social consequences of sexual violence, as well as the effectiveness of interventions. The training of healthcare professionals should be evaluated to ensure more empathetic and comprehensive care. Implementing standardized protocols and care flows can improve the quality and integration of healthcare services, while research on access barriers faced by women can inform interventions that improve accessibility and equity in care. Finally, it is essential to explore strategies to raise awareness of available services, both for victims and health professionals, ensuring that care is welcoming and respectful.

## 6. Conclusions

Female victims of rape violence reported being motivated to seek health services. They pursued support, protection, and orientation on how to proceed after the rape and wanted to understand their rights and duties. Our findings indicate that participants felt a humanized and welcoming conduct from professionals providing care at the teaching hospital.

Although the teaching hospital presented advances regarding laws, rights, technical procedures, psychological assistance, notification, and follow-ups, some challenges in assisting victims of rape remain. Participants raised the idea that some professionals may judge their acts regarding abortion, despite its legality.

Understanding what female victims of SV, especially rape, value when receiving care and what needs to be improved is essential since public policies must meet the demands of this population. Our results may contribute to reorganizing the healthcare network and range of services targeting female victims of rape.

## Figures and Tables

**Table 1:** Characteristics of the participants.

Age	Education Level	Profession	Race	Marital Status	Sexual Orientation	Income (BRL)	Procedure	Gestational Age	Referred from Another Service
**23**	High School	Nursing Technician *	White	Single	Heterosexual	1500.00	Legal Abortion/MVA ***	8 weeks	No
**24**	Incomplete College Education	Administrative Assistant	Black	Single	Heterosexual	1400.00	Clinical **	-	Specialized Police Station for Women’s Support
**31**	Incomplete College Education	Nursing Technician	White	Single	Heterosexual	1300.00	Legal Abortion/MVA	11 weeks	Private Doctor’s Office
**34**	College Education	Nursing Technician	White	Single	Heterosexual	1400.00	Legal Abortion/MVA	6 weeks	No
**22**	Incomplete High School	Bakery Assistant	Black	Single	Heterosexual	1300.00	Legal Abortion/MVA	6 weeks	No
**32**	Incomplete Elementary School	Homemaker	Black	Married	Heterosexual	400.00	Legal Abortion/Pharmacological and Uterine Curettage	19 weeks	Public Primary Healthcare Service
**58**	High School	Nursing Technician	Black	Single	Heterosexual	2000.00	Clinical	-	Police Station

* Nursing technician: In Brazil, this is used to describe individuals who have received specific training and certification to assist in nursing tasks without holding a full nursing degree. ** Clinical assistance refers to prophylaxis of sexually transmitted infections and emergency contraception administration. *** MVA refers to Manual Vacuum Aspiration.

**Table 2:** Positive perceptions, challenges, and possibilities to improve care for female victims of rape.

Positive Perceptions	Challenges	Possibilities
Reference service in the state	Training the healthcare team	Gynecological and psychosocial outpatient follow-up
Motivation to seek health services	Fear of suffering institutional violence	Multi-professional team in the 24 h care service
Support, protection, and orientation	Low adherence to prophylaxis and emergency contraception	Permanent professional education for the healthcare team and managers
Understanding and legitimizing women’s rights		Promotion of the hospital services
Quick and efficient care		
Performance of legal abortion at the hospital		

## Data Availability

The original contributions presented in this study are included in the article. Further inquiries can be directed to the corresponding authors.

## Appendix A. Script Followed in the Semi-Structured Interview

(1) Age
(2) Schooling
(3) Race/color
(4) Marital status
(5) Sexual orientation
(6) Professional occupation
(7) Income
(8) How did you know about the service offered in this hospital?
(9) What motivated you to seek care in this hospital?
(10) How were you welcomed into this service?
(11) Did you receive clarifications on the stages related to the care offered in this hospital? Were you offered the possibility to refuse any procedure or intervention?
(12) Did a multi-professional team provide the care, that is, the care counted with the presence of physicians, nurses, social workers, and psychologists?
(13) Did you feel fear, shame, or embarrassment at any point during the care provided by the health professionals?
(14) Were you offered orientation and education regarding your rights as a woman victim of violence?
(15) Was prophylaxis against sexually transmitted infections and emergency contraception guaranteed? Explain.
(16) Were you offered the possibility of outpatient follow-up?
(17) What possibilities would you list to improve the care for women victims of violence?
(18) What difficulties do you see in the care for women victims of violence?
(19) Do you have any suggestions to improve the services offered in this hospital?
(20) If participants do not spontaneously talk about abortion, as provided by law, questions explored:
  - How did health professionals approach abortion during care?
  - Was there any difficulty in receiving care?
  - Was there any moment you considered the service insufficient? If so, when?
  - What did you consider positive in abortion care?
  - Do you have any suggestions to improve abortion-related care?
